# The Nurses' Attitudes Towards Patients With Non‐Suicidal Self‐Injury: Development and Preliminary Validation

**DOI:** 10.1002/nop2.70188

**Published:** 2025-03-22

**Authors:** Fatemeh Ghaedi‐Heidari, Jahangir Maghsoudi, Masoud Bahrami, Gholamreza Kheirabadi, Bahar Noori‐Rahmatabadi, Alaa Hamza Hermis, Kadhim Hussein Jaasim, Murtadha Abdulridha Ajel, Mohammad Ali Zakeri

**Affiliations:** ^1^ Nursing and Midwifery Care Research Center, Faculty of Nursing and Midwifery Isfahan University of Medical Sciences Isfahan Iran; ^2^ Behavioral Sciences Research Center, Department of Psychiatry, School of Medicine Isfahan University of Medical Sciences Isfahan Iran; ^3^ Social Determinants of Health Research Center Rafsanjan University of Medical Science Rafsanjan Iran; ^4^ Assistant Professor of Anesthesiology and Critical Care Rafsanjan University of Medical Sciences Rafsanjan Iran; ^5^ Al‐Qadisiyah University ‐ Nursing College Al‐Dewaniyah Iraq; ^6^ Nursing College Al‐Mustaqbal University Hillah Babylon Iraq; ^7^ Pistachio Safety Research Center Rafsanjan University of Medical Sciences Rafsanjan Iran; ^8^ Clinical Research Development Unit, Ali‐Ibn Abi‐Talib Hospital Rafsanjan University of Medical Sciences Rafsanjan Iran

**Keywords:** attitudes, nurses, psychometric properties, self‐injurious behaviours, validation

## Abstract

**Aim:**

Non‐suicidal self‐injury (NSSI) is a public health problem throughout the world. Nurses are usually the first caregivers in the mental health team who deal with people with NSSI. Since the consequences of self‐injury are associated with its care and treatment, nurses' attitudes towards these people are an important issue. This study aimed to develop and test a scale to measure nurses' attitudes towards patients with non‐suicidal self‐injury in the Farsi language.

**Design:**

A methodological survey.

**Methods:**

In this methodological study, to scale preparation, psychometric steps were performed on a questionnaire based on the results of a qualitative study and literature review. Exploratory Factor Analysis was conducted to identify the underlying structure of the scale.

**Results:**

The results of the psychometric evaluation showed that the scale included the 22‐item NANSSI in four dimensions, including negativism about the consequences of caring, adopting a counselling approach, violating caring norms, and perceptions about self‐injury. By Cronbach's alpha equal to 0.75, its reliability is estimated. This scale showed a good validity and reliability, which is applicable to evaluate quality of care for patients with non‐suicidal self‐injury.

**No Patient or Public Contribution:**

No patient or public contribution.

## 1 Introduction

Non‐suicidal self‐injury (NSSI) is a common behavioural problem throughout the world (Plener et al. 2015), the prevalence of which is increasing (Karman et al. 2015; Ngune et al. 2021). The prevalence of NSSI varies in different age groups, with adolescents at a higher risk (Stanford et al. 2017). The prevalence of this disorder varies across countries. In western countries, including the USA and Canada, the prevalence of NSSI among adolescents and young adults has been reported to be between 1.50% and 54.80% (Swannell et al. 2014). In non‐western countries, its prevalence among adolescents and young adults ranged from 9% in Japan to 33% in Hong Kong (Gholamrezaei et al. 2017). Several studies have reported its high prevalence in the Iranian population. One study reported that NSSI was seen in 4.30% of the second‐grade high school students in Tabriz (Mohammadpourasl et al. 2008). In another study, the prevalence of NSSI was 12% in female students in the second and third grades of high school in Tehran (Peivastegar 2014). Another study also showed that 8.70% of people referring to forensic settings in Shiraz had a history of NSSI (Gholamzadeh et al. 2017).

In the last decades, NSSI and borderline personality disorder were categorised in the same group of psychiatric disorders (Stead et al. 2019). However, NSSI was considered an independent disorder in the fifth edition of DSM‐V in 2013, which needed further studies (Association 2013). The International Society for the Study of Self‐Injury (ISSS) (2018) defines it as the deliberate, self‐inflicted damage of body tissue without intent to commit suicide and for purposes not socially or culturally sanctioned.

NSSI associates with many mental disorders such as depression and anxiety, post‐traumatic stress (Bentley et al. 2015), substance abuse (Moller et al. 2013), eating disorders (Claes and Muehlenkamp 2016), and suicide (Law et al. 2017). Evidence also suggests that NSSI can predict suicide strongly (Muehlenkamp et al. 2019), as the suicide prevalence in self‐harm people is higher than in those who do not self‐harm (Bolster et al. 2015).

People with experience of self‐harm are more probable to visit the emergency department and psychiatric centres more than once a year (Turner et al. 2014). Frequent hospital admission of people with NSSI can impose an extra cost on the healthcare system and could adversely affect the quality of care (Saunders et al. 2012). Nurses are usually the first line of delivery for psychiatric care who deal with NSSI. Since nurses' attitudes towards patients who self‐harm are an important issue (Siau et al. 2019; Yue et al. 2024), they may affect clinical efficacy and therapeutic‐care outcomes and can prevent further injury and its subsequent consequences like suicides in patients with NSSI (Saunders et al. 2012). For example, nurses with better attitudes can provide the proper information to patients, screen for psychological problems, identify high‐risk suicidal cases, and prevent it and then provide appropriate health care (Dougall et al. 2014). In cases where nurses have inappropriate attitudes, they might behave non‐professionally, such as with hostility, reproach, and non‐empathetic behaviours (Artis and Smith 2013), so it could threaten patient safety (Osafo et al. 2012). Deliberate Self‐Harm Questionnaire (ADSHQ) (McAllister et al. 2002) and Self‐Harm Antipathy Scale (SHAS) (Patterson et al. 2007) were used to assess nurses' attitudes towards patients with NSSI. The *questionnaires correspond with the context of the UK and Australia* and assessed nurses' attitude towards the deliberate self‐harm (DSH). Deliberate self‐harm is a broad concept that encompasses multiple self‐injurious behaviours, including self‐poisoning, whereas in DSM‐V, self‐poisoning has been excluded from the NSSI‐related behaviours (Association 2013). In addition, the type of self‐injurious behaviours can influence nurses' attitudes. Therefore, it is necessary to design an instrument to measure nurses' attitudes towards people with self‐injurious behaviours. In addition, the literature review showed insufficient information about nurses' attitudes towards NSSI patients in Iran. Attitude is a context‐based concept. Considering the importance of nurses' attitudes towards NSSI patients and the lack of proper instruments for measuring nurses' attitudes towards patients with NSSI in Iran, the present study aimed to design a questionnaire influenced by the Iranian culture and to assess the nurses' attitudes towards NSSI.

## 2 Method

### 2.1 Study Design and Setting

This study was the final psychometric section of a sequential exploratory mixed‐methods study. In this methodological study, the scale of Nurses' Attitudes towards Non‐Suicidal Self‐Injury (NANSSI) was developed and validated at Isfahan University of Medical Sciences. Overview of the study is divided into two phases. The first phase was item generation. The second phase was to determine face, content‐construct validity, and reliability.

#### 2.1.1 First Phase: Item Generation

The items pool was constructed from two sources: a qualitative study and published literature on nurses' attitudes towards patients' experiences with self‐injurious behaviours.

First, a qualitative content analysis was carried out to discover staff experiences of (including nurses and physicians) self‐harming clients and their families. According to the content analysis, a 67‐item pool was prepared using the qualitative method. The literature review was conducted to enrich the scale's items. The databases of PubMed, Scopus, Web of Science, and Google scholar were searched from October 1986 to 2017 by Mesh keywords related to validation, attitudes, self‐injurious behaviours, and nurses. After the literature review, pool items expanded by four new items. The 71‐item NANSSI was based on a five‐point Likert scale with an ‘undecided’ midpoint (from one = ‘strongly disagree’ to five = ‘strongly agree’). Negative items were scored inversely (from one = ‘strongly agree’ to five = ‘strongly disagree’). In the final version of the scale, these items were 2, 4–9 and 11–17. The possible range of scores was between 30 and 210. Higher scores indicated a more positive attitude towards the patients with NSSI.

#### 2.1.2 Second Phase: Validity and Reliability

First, 10 nurses working in educational hospitals in Isfahan, Iran, were chosen for the assessment of face validity by Quantitative and qualitative methods. In the qualitative method, the subjects stated their opinions about items' difficulty and ambiguity. By the quantitative method, item impact was scored, and items with scores < 1.5 were omitted from the scale (Shahhosseini et al. 2012).

In the second phase, 16 experts including psychologists (*n* = 2), physicians (*n* = 6), and nursing faculty (*n* = 8) helped us to analyse the contact. Content validity was approved by quantitative and qualitative methods.

In the qualitative method, the suitability, simplicity, and comprehensibility of each item are assessed by the cogent professional comments. So, by these comments, the items were revised or edited. In the quantitative method, the experts were asked to complete the scale based on a three‐point Likert scale (1 = not necessary, 2 = helpful, but not necessary, and 3 = necessary). Quantitative assessment was done based on a three‐point Likert scale (1 = not necessary, 2 = helpful, but not necessary, and 3 = necessary) by experts. *According to* the *Lawshe table, when the total number of experts is 16*, *the cut*‐*point value would be 0.49* (Lawshe 1975). The relevancy, simplicity, and clarity of each item and the scale (Content validity index = CVI) were evaluated and graded based on a four‐point Likert scale (ranging from one to three for relevancy, ranging from one to four for simplicity, and ranging from one to four for clarity). Based on the literature review, 0.9 and 0.80 or higher are the acceptable standard values of I‐CVI and S‐CVI, respectively (Zamanzadeh et al. 2014).

In the third phase, a preliminary item analysis was also conducted. The reliability of the overall scale and inter‐item correlations were calculated by 50 subjects: nurses who were working in the educational hospitals in Isfahan, Iran.

Fourth, construct validity was assessed. Also, the reliability of the overall scale and each subscale was calculated by 200 subjects: nursesworking in educational hospitals in Isfahan, Iran. Socio‐demographic data such as age, gender, marital status, educational level, work experience, and working ward were assessed. The data from 185 nurses were included in the final analysis.

### 2.2 Data Analysis

Using the exploratory factor analysis (EFA), principal component analysis (PCA), and varimax rotation, we could evaluate the underlying variable: attitudinal structure. A sample size less than the minimum 5:1 subjects‐to‐item ratio necessary for exploratory factor analysis was considered. Nevertheless, the Kaiser–Meyer–Olkin (KMO) measure of sampling adequacy index was 0.88, and the Bartlett's test of sphericity was significant (*χ* ^2^ = 1400.93, *p* < 0.001). These results indicated that the sample size and data were adequate for conducting an exploratory factor analysis. EFA showed that the sample and correlation matrix were within an acceptable range. The following criteria were used to identify the number of factors: (a) the number of components with an eigen value > 1, (b) the Scree Plot, and (c) interpretability. 0.4 was assumed as the minimum load factor. Floor and ceiling effects would have not occurred if fewer than 15% of participants' scores cluster towards the worst and best scores.

Internal consistency approves reliability of the questionnaire if Cronbach's alpha was equal or greater than 70%. Missing value analysis was performed. If 20% of the answers of a question were clear or vague that question would be removed. Analysis of the deleted questions was done by ‘case mean substitution’ method. Using Statistical software: SPSS Ver‐ 19.0, data was analysed. For all the tests, a *p* value < 0.05 was assumed as a cut off to mean a significant difference.

### 2.3 Ethical Considerations

Isfahan University of Medical Sciences (IUMS) approved this project (code of ethics = IR.MUI.REC.1395.3.943). Then, we informed subjects about the study purpose, the confidentiality of the data, and participants' anonymity. The subjects were assured that they were allowed to withdraw freely from the study at any time. So, they consented to participate in this study via signing a form.

## 3 Results

### 3.1 Face Validity

According to nurses' views, 10 items were simplified. In 10 items ‘impact scores’ was below 1.5. The item impact scores ranged from 1.5 to 4.9. Based on item impact scores and the importance of each item, the research team decided to remove one item. Using the face validity tests, content validity was evaluated by 61 items.

### 3.2 Content Validity

According to experts' comments, six items were vague, not comprehensive, and had considerable conceptual overlap, so they were omitted or merged into other items. Content Validity Ratio (CVR) values of 11 items were lower than the paired values of the Lawshe table (0.49). Also, in calculating the Content Validity Index (CVI), 8 items with scores < 0.78 were eliminated. By the content validity evaluation, the scale contained 32 items.

### 3.3 Preliminary Item Analysis

Before performing EFA, the internal consistency of the total scale was determined. The Cronbach's alpha of the total scale was 0.75. In order to the Cronbach's alpha value 10 items with negative item‐total correlation were omitted, and the internal consistency recalculated, so the alpha value became 0.85. The initial reliability analysis showed a range of correlations suggesting that a factor analysis including 22 items would be appropriate (Table 1).

**TABLE 1:** Item analysis before EFA.

Original no.	Cronbach's alpha if item is deleted	Modified item‐total correlation
3	0.37	0.84
4	0.22	0.85
6	0.45	0.84
8	0.52	0.84
11	0.62	0.83
12	0.46	0.84
13	0.70	0.83
14	0.49	0.84
15	0.33	0.84
16	0.58	0.83
17	0.31	0.84
18	0.39	0.84
19	0.48	0.84
20	0.58	0.83
21	0.36	0.84
23	0.23	0.84
24	0.42	0.84
28	0.29	0.84
29	0.27	0.84
30	0.27	0.84
31	0.26	0.84
32	0.20	0.85

### 3.4 Characteristics of the Population Study

The mean age of nurses was 34.20 (7.54) years. Most nurses were female (81.10%), married (61.20%), had a bachelor's degree (92%), had work experience of 10 years (14.40%) and were employed in the general emergency department (45.50%). The demographic characteristics of subjects are presented in Table 2.

**TABLE 2:** The baseline characteristics of the participants.

Variable	*N* (%)
Gender	
Female	150 (81.1%)
Male	35 (18.9%)
Marital status*	
Single	69 (38.8%)
Married	109 (61.2%)
Educational level*	
Bachelor's degree	162 (92%)
Master's degree	13 (7.4%)
Ph.D degree	1 (0.6%)
Work experience (year)*	
< 10	102 (58.6%)
10–20	59 (33.9%)
> 20	13 (7.5%)
Working ward*	
Surgical	69 (38.8%)
General emergency	81 (45.5%)
Psychiatric emergency	7 (3.9%)
Psychiatric	21 (11.8%)
	Mean (SD)
Age	34.20 (7.54)

Abbreviation: SD, Standard deviation.

*Missing data.

### 3.5 Construct Validity

Construct validity was approved by EFA. The second table of the exploratory factor analysis included the values associated with the initial eigenvalues and extraction subscriptions. At this stage, the minimum factor load was 0.4. Therefore, all items were appropriate and applied. The calculated extraction subscription values of all the items analysed by exploratory factor were between 0.42 and 0.79.

**FIGURE 1:**
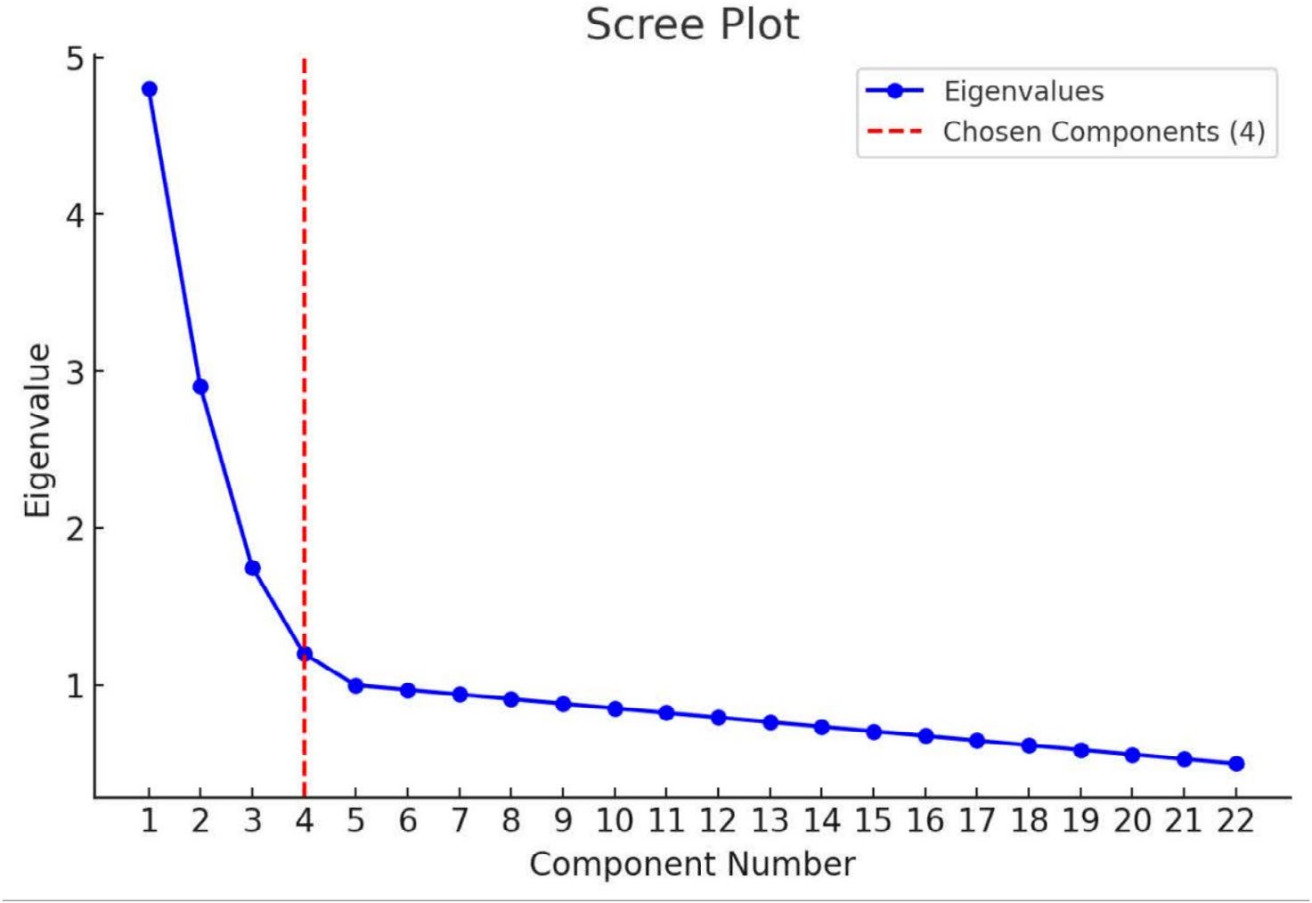
Scree plot.

The third table of exploratory factor analysis included a special value of extraction factors without rotation, a special value of extraction factors with rotation, and initial eigenvalues. Eigenvalues > 1 were used to estimate the number of factors, indicating a seven‐factor solution with a predictive power of 64.76. However, if the seven‐factor solution were meaningless or less parsimonious, the screen test of eigenvalues would be plotted against it. This test suggested that only five to six factors should be retained. As part of parallel analysis, the Monte Carlo PCA program (Watkins 2000) was carried out to compare the size of eigenvalues obtained from a randomly generated data set of the same size. The results suggest that four factors should be retained. Four factors had a predictive power of 48.97% (Table 3). Parallel analysis enabled us to provide an accurate number of factors and helped distinguish important factors from trivial ones (Ledesma and Valero‐Mora 2007). Four meaningful subscales emerged: negativism about the consequences of caring (Factor 1) with 7 items, adopting a counselling approach (Factor 2) with 5 items, violating caring norms (Factor 3) with 5 items, and perceptions of self‐injury (Factor 4) with 5 items. Also, the scree plot was used to determine the number of extractable factors. Accordingly, 4 extractable factors were observed (Figure 1).

**TABLE 3:** Initial and extraction subscriptions of exploratory factor analysis of NANSSI.

Component	Rotation sums of squared loadings		
	Total	% of variance	Cumulative %
Negativism about the consequences of caring	3.25	14.35	14.35
Adopting a counselling approach	3.09	13.69	28.04
Violating caring norms	2.61	13.34	41.39
Perceptions of self‐injury	1.93	7.58	48.97

Table 4 shows the rotated component matrix, which included the factor loads of the variables in the remaining factors after the rotation.

**TABLE 4:** Rotated component matrix for NANSSI.

Original no.	Item	F1	F2	F3	F4
3	The patient with NSSI is disappointed to be supported by others				0.75
4	The patient with NSSI suffers from a kind of brief psychosis				0.52
6	The patient with NSSI may be a victim of some socioeconomic problems				0.61
8	The patient with NSSI does not have strong religious beliefs				0.49
11	The patient with NSSI is an extra burden on the healthcare system	0.50			
12	It is a waste of time to communicate with the patient with NSSI.	**0.75**			
13	I do not want to take care of a patient with NSSI	**0.75**			
14	I had to tolerate a patient with NSSI	0.69			
15	I hate the patient with NSSI	0.66			
16	I feel sympathetic for a patient with NSSI	0.54			
17	I am afraid of a patient with NSSI because she/he may hurt me physically	0.50			
18	The problems of a patient with NSSI come from his/her immature personality				**0.83**
19	A patient with NSSI is not competent in his/her life			0.79	
20	A patient with NSSI is not reliable			**0.88**	
21	A patient with NSSI does not commit to any relationship			0.73	
23	It is difficult to have a fair behaviour towards a patient with NSSI			0.71	
24	The patient with NSSI must be punished to prevent frequent self‐injurious behaviours			0.59	
28	Specific counselling skills are needed to deal with a patient with NSSI		**0.80**		
29	The patient with NSSI must be accepted and perceived		0.79		
30	The patient with NSSI must receive more counselling services for more accurate evaluation and better treatment		0.78		
31	The patient with NSSI must receive information about social support sources		0.69		
32	A patient with NSSI can learn new coping strategies		0.65		

*Note:* F1, Negativism about the consequences of caring; F2, Adopting a counselling approach; F3, Violating caring norms; F4, Perceptions of self‐injury. Bold values shows the highest amount in each F.

### 3.6 Reliability

Cronbach's alpha coefficient values were 0.76, 0.65, 0.78, 0.69, and 0.75 for the total scale, subscales of negativism about the consequences of caring, adopting a counselling approach; violating caring norms; and perceptions of self‐injury, respectively.

## 4 Discussion

The current study aimed to design a NANSSI to assess nurses' attitudes towards self‐injury, and its validity and reliability. The instrument consisted of 22 items and 4 subscales include ‘negativism about consequences of caring’, ‘adopting the counseling approach’, ‘violating caring norms’, and ‘perceptions of self‐injury’. Based on the results of this study, NANSSI is considerably valid and reliable for nurses' attitude assessment. According to the literature review, ADSHQ designed by McAllister et al. in Australia McAllister et al. (2002), and SHAS designed by Patterson et al. in the UK Patterson et al. (2007) were used to assess nurses' attitudes towards people who self‐harm but both instruments were designed more than 10 years ago, and the socio‐cultural conditions of countries are dynamically changing, which can affect nurses' attitudes towards people with NSSI. Therefore, NANSSI can be introduced as a new Persian instrument for nurses' attitudes towards patients with NSSI. In addition, the ADSHQ items were designed based on interviewing with several emergency students and the literature review. Nurses' experience of patients with NSSI in psychiatric wards is invaluable to design the relevant instrument, but it has been ignored. Unlike the ADSHQ, various sources have been used to design SHAS items, but it is controversial how to use articles published on attitudes towards suicidal behaviour as a source of item generation. Therefore, the SHAS items do not purely reflect the nurses' attitudes towards patients with NSSI. However, the questionnaire designed in the present study is the first instrument, which items have been drawn from multiple sources including qualitative interviews with nurses working in different wards (surgery, emergency and psychiatry), physicians, psychologists, and patients with NSSI and their families as well as literature review. Multiple sources can make NANSSI items more comprehensive than ADSHQ and SHAS. In addition, NANSSI only measures nurses ‘attitudes towards people with NSSI, while the two other instruments measure nurses’ attitudes towards patients with DSH. Deliberate self‐harm encompasses both suicidal and non‐suicidal self‐injurious behaviours, while NSSI includes only non‐suicidal self‐injurious behaviours (Karanikola et al. 2018). The conceptual differences between deliberate self‐harm and NSSI can influence nurses' attitudes.

In the present study, ‘negativism about consequences of caring’ in the NANSSI indicates that patients with NSSI are perceived as an extra burden on the healthcare system, which makes nurses reluctant to take care of them. This subscale can be related to the uncontrollable condition of the patient (Sandy and Shaw 2012) because NSSI is recurrent in nature (Larkin et al. 2014) and one may think that the healthcare team is unable to control the disease. In addition, continuous monitoring of patients with self‐injurious behaviours imposes a high burden on nurses physically and mentally (Favara 2013; Wilstrand et al. 2007), which can reduce nurses' willingness to providing care to patients with NSSI. The items of this subscale of NANSSI correspond with the negative ones in the subscales: ‘dealing effectively with DSH clients’ in ADSH and ‘the care futility’ in SHAS.

Based on the results of the study, the subscale ‘adopting a counseling approach’ focuses on the importance of applying therapeutic communication techniques (such as listening and providing leads), person‐centered communication techniques (such as empathy) and mental empowerment (such as training coping skills). According to Inkle, self‐harm can be a way of adjustment and nurses who are aware of it exhibit more counselling behaviours such as empathy than other nurses do (Gonzales and Bergstrom 2013). Also, according to a study, nurses with empathetic behaviours accept and respect the patient and believe that the patient is a unique and they empower patients through coping skills training (Slater et al. 2017). This subscale of NANSSI corresponds with the ‘empathic approach’ and ‘dealing effectively with DSH clients’ in ADSH, ‘acceptance, understanding, and competence appraisal’ in SHAS.

The third subscale of NANSSI is ‘violating caring norms’ that includes behaviours such as labeling and punishing patients with NSSI. Offensive and inhumane labels can be a sign of nurses' misunderstanding of patients' motivations for injurious behaviours (Shaw and Sandy 2016). In addition, labeling does not establish a communication based on respect and value (Ramluggun 2013). In addition, it predisposes to discriminate between these patients and other ones (Law et al. 2009). These results indicate that nurses' negative attitudes cause non‐professional behaviours such as punishing NSSI patients. The experiences of some NSSI patients show that punishment in the psychiatric services, including nursing services and care, lead to sense of isolation and low self‐esteem (Horrocks et al. 2005). The items of this subscale of the questionnaire do not correspond with ‘acceptance and understanding’ in SHAS and any subscales of ADSHQ. Such a subscale could be related to the nurses' poor knowledge of NSSI patients, which is similar to the results of other studies (Conlon and O'Tuathail 2012; Saunders et al. 2012; Timson et al. 2012; Vine et al. 2017; Wheatley and Austin‐Payne 2009).

The fourth subscale is ‘nurses' perceptions of self‐injury’ including the nature, functions, and risk factors of self‐injury. The results of the study showed that some nurses are misunderstood about the nature of NSSI so that considered it a brief psychosis. This result supports the study of Warm et al. (2003), which considered self‐injury a symptom of brief psychosis (Warm et al. 2003). According to Lloyd‐Richardson et al. (2007) theoretical model, ‘performance’ refers to the immediate antecedents leading to NSSI that reinforce self‐injurious behaviours and its frequency (Lloyd‐Richardson et al. 2007). Patients with NSSI use their body language because of poor communication skills. People self‐harm when they do not establish verbal communication with others and want to achieve their desires (Machoian 2001; Potter 2003). Being disappointed to receive help from others is an example of self‐injury mentioned in the questionnaire items. In addition, based on the results of the study, NSSI has several risk factors with psychological, spiritual and social dimensions. Immature personality in the items is one of the causes of NSSI. Several studies have also shown that low self‐control and high irritability are the factors associated with NSSI (Brent et al. 2013; Gallinat et al. 2019; Ghaedi Heidari et al. 2019). In addition, several studies have shown the relationship between histrionic personality disorder and NSSI (Ghaedi Heidari et al. 2019; Krishnaram et al. 2016; Vaughn et al. 2015). In addition, Latina and Stattin (2018) (Latina and Stattin 2018) and Junker et al. (2019) (Junker et al. 2019) showed self‐injurious behaviours in adolescents with poor self‐esteem. Patients' mental health problems are also at the risk of NSSI. This factor has been neglected in many relevant textbooks, but most Iranian people are Muslim, and it is expected from researchers to consider this factor more carefully. A meta‐analysis showed that spirituality and religion predicted individuals' mental health and if individuals were deeply religious, they would increase mental health. Since self‐harm is a symptom of poor mental health, it can indicate spiritual problems in patients with NSSI (Smith et al. 2003). Contrary to this result, a study showed no association between spirituality and NSSI (Good et al. 2017). Based on the results of the present study, socioeconomic problem is another risk factor for patients with NSSI. This subscale of NANSSI corresponds with ‘the perceived confidence in the assessment and referral of DSH clients’ in ADSHQ, ‘client intent manipulation, acceptance, and understanding’ in SHAS.

### 4.1 Limitations

The strengths of our study include the validation of a questionnaire for assessing *Nurses' Attitudes towards Patients with Non‐Suicidal Self‐Injury* among the Iranian population. Our study was conducted in central Iran, and caution is necessary when interpreting the results. Factors such as culture, socioeconomic status, and education, which may vary across different regions, can influence the outcomes. Therefore, further investigation in larger and more diverse groups is required.

## 5 Conclusion

NANSSI exploring Iranian nurses' attitudes to NSSI patients identified four subscales and showed acceptable validity and reliability. Therefore, this scale is applicable in such research, but conclusions at this stage are tentative because of the study limitations, including the use of a non‐random sample and the reliance on self‐report data. The first issue makes generalisation difficult. The second issue (self‐report) may result in non‐real scores because it is possible their real views conflict with their professional self‐image.

## Author Contributions

F.G.‐H., J.M., and M.B. designed the study. F.G.‐H. and G.K. analysed data collection. F.G.‐H., B.N.‐R., A.H.H., and M.A.Z. drafted the manuscript. M.A.Z., A.H.H., K.H.J., M.A.A., and M.B. revised the manuscript for intellectual content. All authors read and approved the final version.

## Conflicts of Interest

The authors declare no conflicts of interest.

## Supporting information


Data S1.

## Data Availability

The data that support the findings of this study are available from the corresponding author upon reasonable request.
